# MOCVD Growth of High-Quality and Density-Tunable GaAs Nanowires on ITO Catalyzed by Au Nanoparticles Deposited by Centrifugation

**DOI:** 10.1186/s11671-015-1121-y

**Published:** 2015-10-20

**Authors:** Dan Wu, Xiaohong Tang, Ho Sup Yoon, Kai Wang, Aurelien Olivier, Xianqiang Li

**Affiliations:** OPTIMUS, Photonics Centre of Excellence, School of Electrical and Electronic Engineering, Nanyang Technological University, 50 Nanyang Avenue, Singapore, 639798 Singapore; Division of Structural Biology & Biochemistry, School of Biological Sciences, Nanyang Technological University, 50 Nanyang Avenue, Singapore, 639798 Singapore; Department of Electrical & Electronic Engineering, South University of Science and Technology of China, 1088 Xueyuan Avenue, Shenzhen, 518055 China; CINTRA UMI 3288, School of Electrical and Electronic Engineering, Nanyang Technological University, Research Techno Plaza, 50 Nanyang Drive, Border X Block, Level 6, Singapore, 637553 Singapore

**Keywords:** GaAs nanowires, Indium tin oxide, Centrifugation, MOCVD

## Abstract

High-quality and density-tunable GaAs nanowires (NWs) are directly grown on indium tin oxide (ITO) using Au nanoparticles (NPs) as catalysts by metal organic chemical vapor deposition (MOCVD). Au catalysts were deposited on ITO glass substrate using a centrifugal method. Compared with the droplet-only method, high-area density Au NPs were uniformly distributed on ITO. Tunable area density was realized through variation of the centrifugation time, and the highest area densities were obtained as high as 490 and 120 NP/μm^2^ for 10- and 20-nm diameters of Au NPs, respectively. Based on the vapor–liquid–solid growth mechanism, the growth rates of GaAs NWs at 430 °C were 18.2 and 21.5 nm/s for the highest area density obtained of 10- and 20-nm Au NP-catalyzed NWs. The growth rate of the GaAs NWs was reduced with the increase of the NW density due to the competition of precursor materials. High crystal quality of the NWs was also obtained with no observable planar defects. 10-nm Au NP-induced NWs exhibit wurtzite structure whereas zinc-blende is observed for 20-nm NW samples. Controllable density and high crystal quality of the GaAs NWs on ITO demonstrate their potential application in hybrid a solar cell.

## Background

Semiconductor nanowires (NWs) have been intensively explored due to their outstanding potential as the building blocks for future photonic and electronic devices [1, 2]. NWs-based solar cells [3, 4] and field-effect transistors (FETs) [5] have been realized on the basis of NWs’ superior optical and electrical properties including relaxation of lattice strain and capability for top-down and bottom-up assembly etc. [6]. The vapor–liquid–solid (VLS) mechanism [7] describes that the vapor phase growth precursors form supersaturated liquid alloys inside Au nanoparticles (NPs) at elevated temperatures [8], and the chemical potential difference drives the precipitation of semiconductor material to the liquid–solid interface, and by continuous supply of the growth material, the eutectic alloys crystallize and form the NWs. NWs growth use Au NPs as catalysts via VLS mechanism by metal organic chemical vapor deposition (MOCVD) is one of the most common and promising techniques for III-V NWs growth because of the precise control over complex axial structures and large-scale commercialization capability.

Among various types of NWs, III-V semiconductor materials such as GaAs are of particular interests owing to direct band gap, high electron mobility [9], and the ability of integration on large lattice mismatch low-cost substrates [8]. High crystal quality GaAs NWs of perfect zinc-blende structure with no planar structural defects were grown directly on soda lime glass [10]. It was also reported that 10-nm GaAs NW-based FET was fabricated on SiO_2_/Si substrate with pure phase wurtzite structure [11]. However, the insulating nature of those low-cost substrates limits further applications. Indium tin oxide (ITO) coated glass as one of the most widely used transparent conductive oxide substrates are therefore investigated as an attractive platform for NWs growth. Hybrid solar cells were designed on the basis of successful growth of InP NWs on ITO for the first time in 2008 [12]. Free-standing GaAs NWs with large tilt angels with ITO surface were demonstrated by our group last year [13]. Despite successful direct integration of III-V NWs on ITO, there are limited reports on the control over the growth rate, density, and crystal quality, which are vital for device design and fabrication.

Together with growth condition, the size of Au catalysts has great influence on the crystal quality of the NWs whereas the distribution of Au NPs determines the area density. Therefore, various Au NP deposition methods are well investigated in order to control NW growth. Normally, the Au NPs are deposited by droplet-only method. The aqueous Au NP solution is dropped onto the substrate and then nitrogen is used to blow away the excess solution, leaving the Au NPs. The particle size distribution is restricted by the Au NP solution synthesis method (typically about 15 %). This droplet-only method is simple and cost-efficient, but the area density of the Au NPs is low, around 0.5 NP/μm^2^ [14]. Annealing a layer of several nanometers of Au thin film is also a popular method to generate Au NPs with area density up to 80 NP/μm^2^ but with a broad particle size distribution [15, 16]. Moreover, the high annealing temperature can reduce the transmittance of ITO. Other examples include depositing Au NPs through nanochannel alumina templates [17], manipulating Au NPs using atomic force microscopy [18] etc. However, these methods are expensive, and the process is rather complicated. Early this year, a cost-effective centrifugal deposition method for Au NPs on ITO was reported where the densities could be rationally controlled by tuning centrifugation time and speed [19]. However, there have been no reports which adopt this promising method for NWs growth.

In this study, we use the centrifugal method to deposit Au NPs on ITO for growing high-quality and density-controllable GaAs NWs by MOCVD. 10- and 20-nm diameter Au NPs were deposited on ITO with fixed effective gravitational force generated by centrifugation whereas the area densities of the Au NPs were tuned through varying the deposition time. Uniform and free-standing with controllable area density GaAs NWs on ITO were grown by MOCVD induced by the Au NPs. The crystal quality of the NWs was evaluated using electron microscopy, which demonstrate nearly planar defect-free wurtzite and zinc-blende structure for the 10- and 20-nm Au NP-catalyzed GaAs NWs. These results demonstrate effective control of GaAs NWs by Au NPs centrifugal method and show possibility for hybrid solar cell application.

## Methods

The GaAs NWs were directly grown on ITO glass substrate via VLS mechanism using Au NPs as the catalysts by MOCVD. Commercial ITO glasses with a 155 (±20) nm-thick ITO layer (Xinyan Technology co., LTD.) were used as the substrates which were cleaned by successively soaking in an ultrasonic bath filled with acetone, isopropanol, and deionized water for 10 min each and dried with N_2_. Polydiallyldimethylammonium chloride (PDDAC) solution (Polysciences Inc. 1 wt.%) was then used to functionalize the surface of ITO substrates for 60 min followed by rinsing with deionized water for 30 s and drying for 10 s. It is also reported that PDDAC soaking forms a positive charged layer which helps attract the negative charged Au NPs onto the ITO surface [20]. Comparative study has proved that PDDAC is more effective in helping attract Au NPs on ITO than commonly used poly-L-Lysine (PLL) [13]. 10- and 20-nm diameter aqueous Au NPs solution (Nanocs Ltd.) with the density of ~1.8 × 10^13^ NP/mL were used in this study. Samples were then completely dried with N_2_ and baked at 100 °C for 3 min before transferred into the MOCVD reactor (Aixtron 200) for the GaAs NWs growth. Trimethylgallium (TMGa) and tertiarybutylarsine (TBAs) were used as metalorganic precursors, and purified H_2_ gas was used as the carrier gas with dew point below −100 °C. The chamber pressure of the reactor was 50 mbar. During the temperature ramping up, TBAs were introduced into the reactor with a molar flow rate of 12.92 μmol/s whereas TMGa was input with a molar flow rate of 1.36 μmol/s when the growth temperature of 430 °C was reached. The V/III ratio was kept as 9.5, and the growth time was fixed at 2 min. LEO 1550 Gemini field emission scanning electron microscopy (FESEM) was used to observe the Au NPs distribution and the morphology of the GaAs NWs grown on ITO substrate. Transmission electron microscopy (FEI Tecnai G20 F20 field emission TEM, operated at 200 kV at room temperature) was used to characterize the crystal quality of the GaAs NWs.

## Results and Discussion

### Deposition of the Au Nanoparticles by Centrifugation

**Fig. 1:**
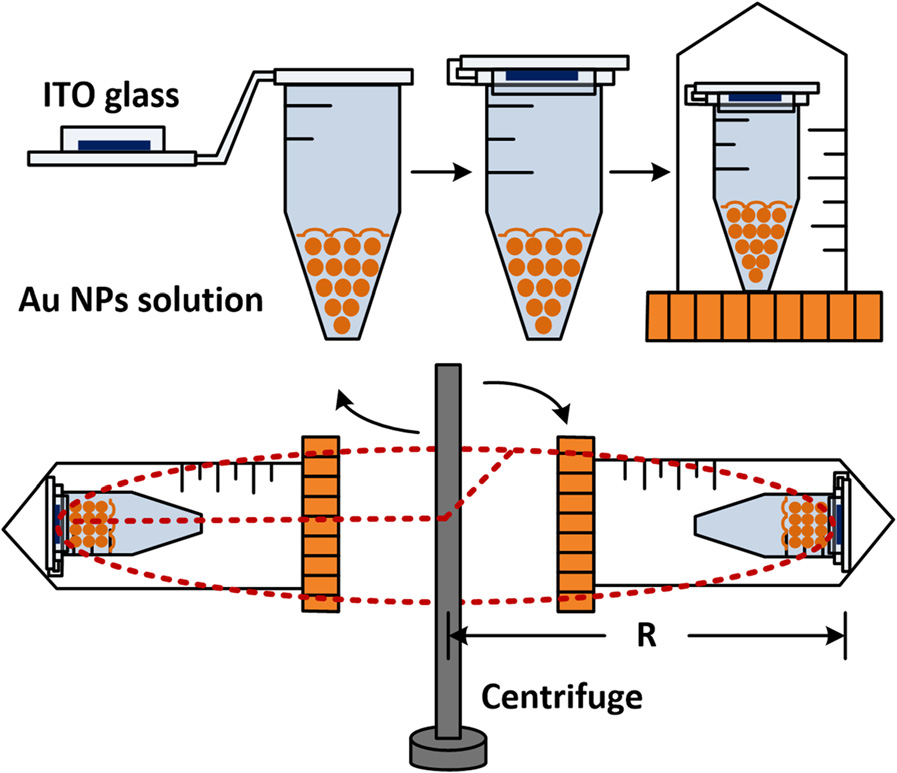
Schematic for Au nanoparticle (NP) centrifugation process

The schematic of Au NPs deposition by centrifugation is illustrated in Fig. 1. After thorough cleaning and functionalization, the 4 × 4-mm ITO substrates were stuck onto a tube cap, and the aqueous Au NPs solution of 0.5 mL was filled in the tube. The tightly sealed tube was transferred into a centrifuge tube which matched the size of the centrifuge (Sartorius 2–5). The radius of the rotor *R* was 13.4 cm, and the relative centrifugal force was calculated by rotation speed revolutions per minute (RPM) using 1.12 × *R* × (RPM/1000)^2^. The centrifugation speed was set at 3900 RPM corresponding to 2283 g, and the centrifugation time changed from 1 to 16 min. As a comparison, a control group was prepared with the same Au NP-solution concentration and the droplet deposition on ITO glass lasted for 3 h. Before inspection under FESEM, all of the samples were dried with N_2_ and baked at 100 °C for 3 min. In the following, area density is defined to describe the number of Au NPs per square micrometer.

**Fig. 2:**
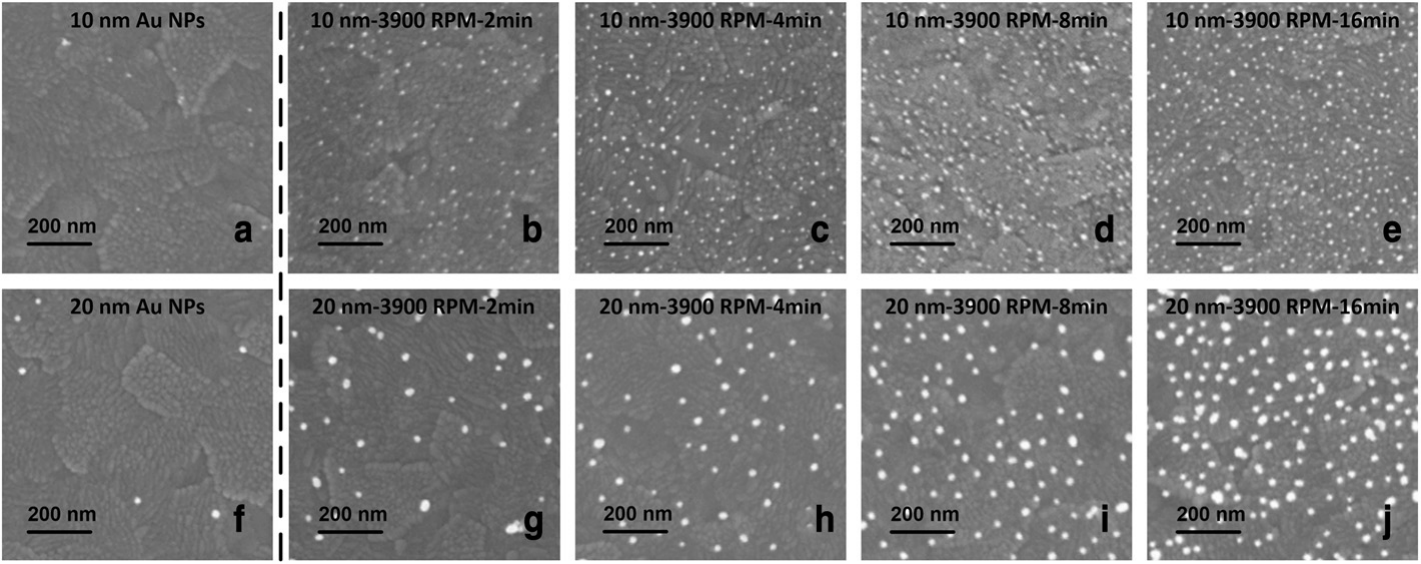
FESEM images of the Au NP distribution on the ITO with different centrifugation time. **a**–**e** are for the 10 nm and (**f**–**j**) are for 20-nm diameter Au NPs. **a**, **f** are droplet-only samples

**Fig. 3:**
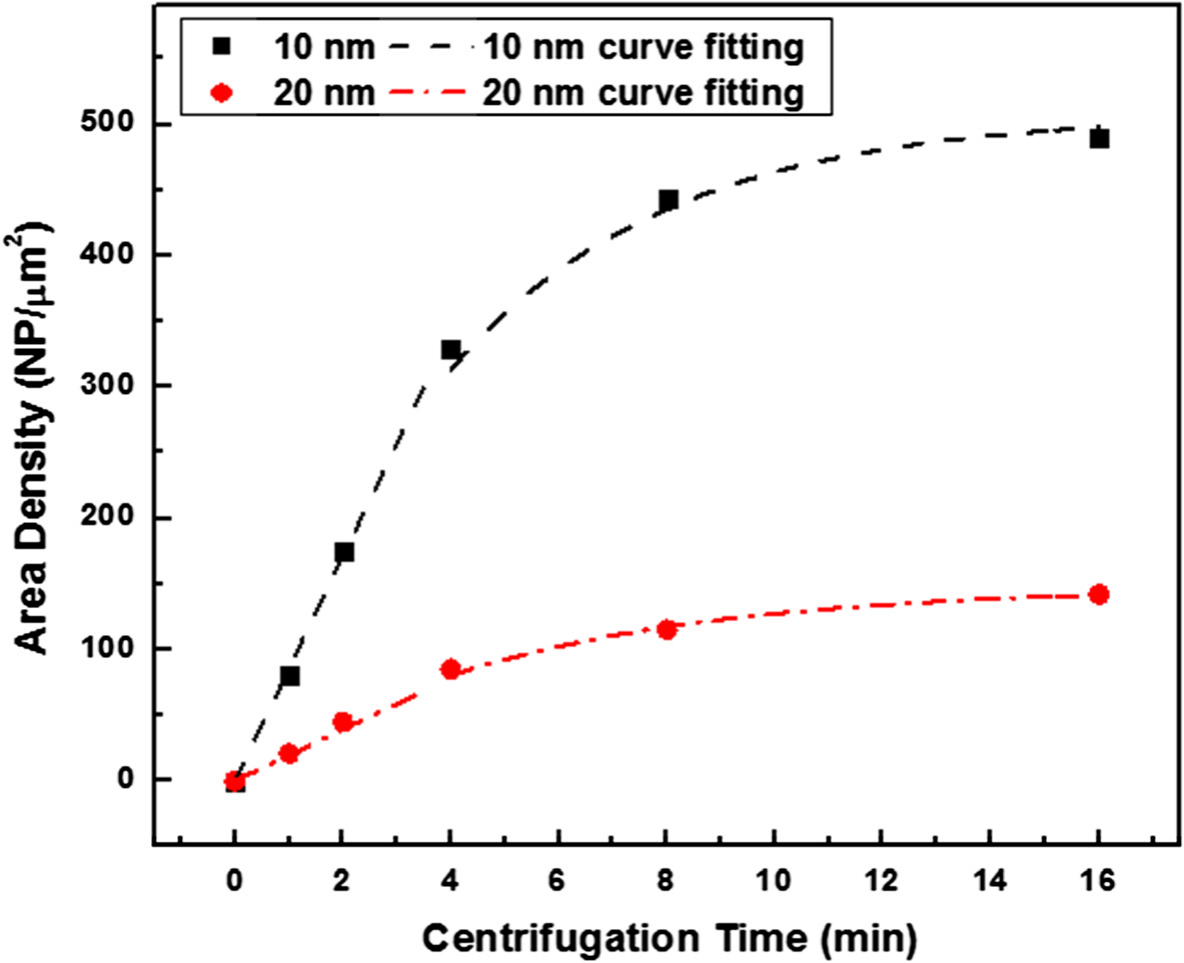
Area density changes with respect of centrifugal time for 10- and 20-nm diameter Au NPs

FESEM images of the 10- and 20-nm Au NPs deposited on ITO by the centrifugation and droplet-only method with various deposition time are shown in Fig. 2. The area densities of both 10- and 20-nm Au NPs deposited on the ITO were about 10 NP/μm^2^ using the droplet-only method. Much higher density of the Au NPs were found on ITO substrates using the centrifugation method. As the centrifugation time prolonged, the area density of both 10- and 20-nm Au NPs rose accordingly. Figure 3 displays the increase trend of both 10- and 20-nm Au NP area density on the ITO changing with the centrifugation time. The fitting curves were calculated based on the equations in reference [19] indicating that the area density approaching saturation when the centrifugation time was 16 min and the highest area densities reached 490 NP/μm^2^ and 120 NP/μm^2^ for 10- and 20-nm Au NPs, respectively. Compared with droplet-only method, the area density can be rationally controlled and greatly increased.

### Growth of GaAs Nanowires on ITO by MOCVD

**Fig. 4:**
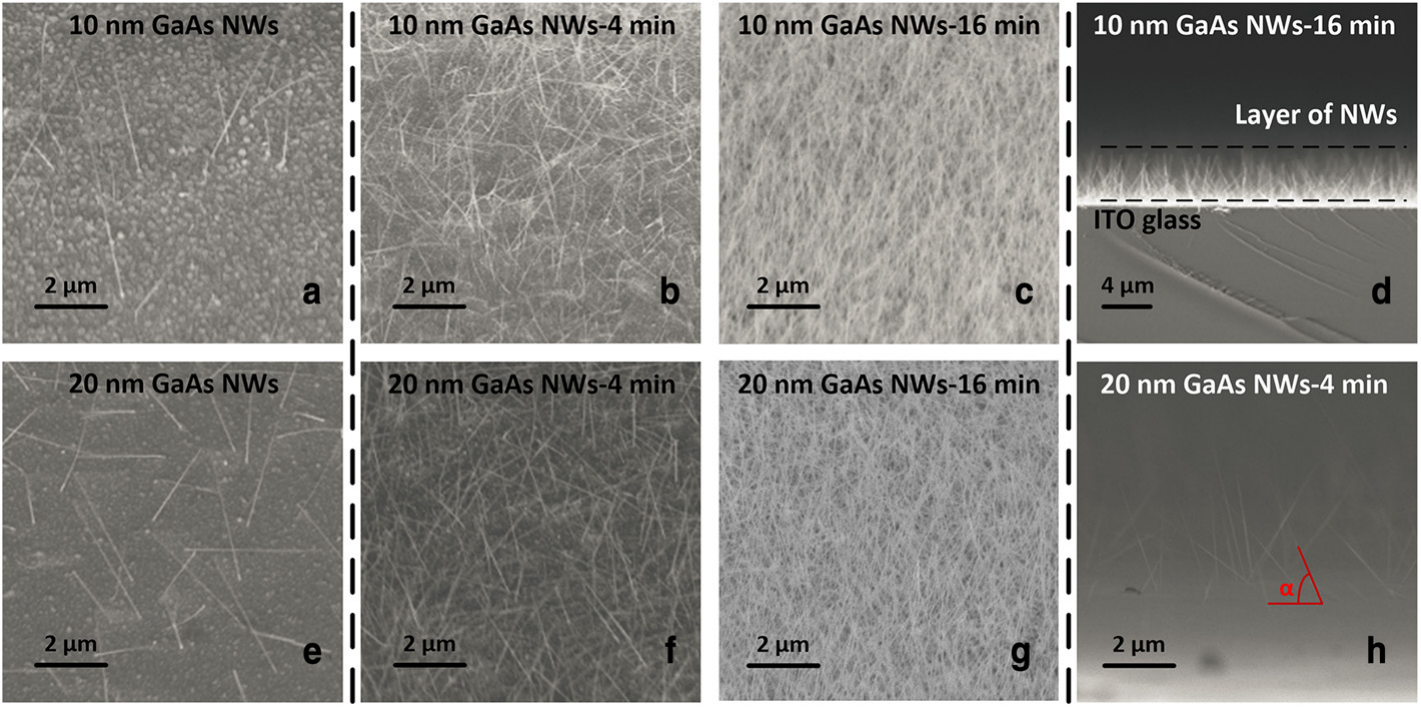
FESEM images of the 10- and 20-nm Au NPs-catalyzed GaAs nanowires (NWs) with different Au NP deposition method. **a** and **e** Droplet-only method, and the rest are centrifugal method. **d** and **h** Cross sectional views of the GaAs NWs

Figure 4 shows the FESEM images of the GaAs NWs grown on ITO catalyzed by the 10- and 20-nm Au NPs deposited by the centrifugal method. Straight and elongated GaAs NWs were received, and there were no observable kinks or worm-shaped defects for both sizes of the NWs. Due to the polycrystalline nature of the ITO, no single particular growth direction of the grown NWs was favored, but most of the NWs stood with large tilt angle *α* with the ITO surface as shown in the cross sectional view Fig. 4h. As shown in Fig. 4b, c, f, and g, following the area density of the Au NPs, high-density GaAs NWs were obtained for both 10- and 20-nm Au NP-catalyzed samples. High density of the NWs are desirable in high-efficiency hybrid solar cell application where the spacing of the NWs should be on the scale of diffusion length of excitons [21].

**Fig. 5:**
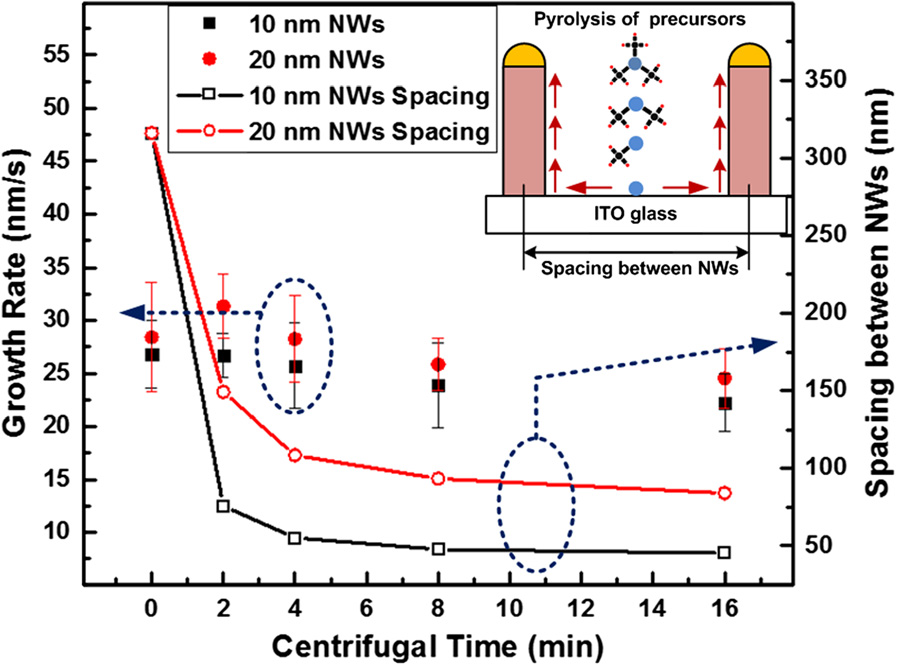
GaAs NW growth rate and spacing between NWs as a function of centrifugal time. The *inset* schematic explains the spacing of NWs and competition of the available adatoms

The growth rate was investigated as a function of centrifugal time as shown in Fig. 5. Since the relationship between centrifugal time and area density of the Au NPs were calculated in Fig. 3, the NW growth rates could be studied with the area density variation. Each data in the figure were calculated from 25 GaAs NWs of the same sample, and the standard deviation was plotted as the error bar. At the same time, NWs spacing changes with centrifugal time is also illustrated in the same figure. The NWs spacing *L*_*NW*_ was introduced to describe the spacing between the adjacent NWs as illustrated in the inset of Fig. 5. The *L*_*NW*_ was calculated as $1000/\sqrt{Area\kern0.5em  Density}$ with the unit of nanometer using the area density values in Fig. 3. In general, the NWs growth rates decrease with the rise of centrifugal time. This is mainly due to the competition of the available adatoms. Based on the kinematic model of NWs growth [22], there are two sources of adatoms for the NWs growth, including direct impingement upon the Au NPs and diffusion along the side wall of the NWs. Under the same MOCVD growth condition (temperature and precursor flow rates), the pyrolysis efficiency of precursors should be the same for growing all the samples leading to the same impingement rate. Therefore, the decrease in the growth rates with the higher area density is attributed to the amount of adatoms available for diffusion along the NWs. When the NWs spacing is larger than the adatoms’ diffusion length, all adatoms on the substrate surface within the diffusion length can contribute to the NWs’ growth. However, when the NW spacing is less than the adatoms diffusion length, the arriving adatoms will be shared among the grown NWs. Under the growth conditions, the diffusion length for Ga adatoms is about 100 nm [23] which is longer than the GaAs NWs spacings for both the 10- and 20-nm Au NPs deposited using the centrifugal method with the centrifugal time of 4 min. Therefore, a sharp reduction of the NWs growth rates was observed for the GaAs NWs growth when the Au NPs were deposited with 4 min centrifugation.

**Fig. 6:**
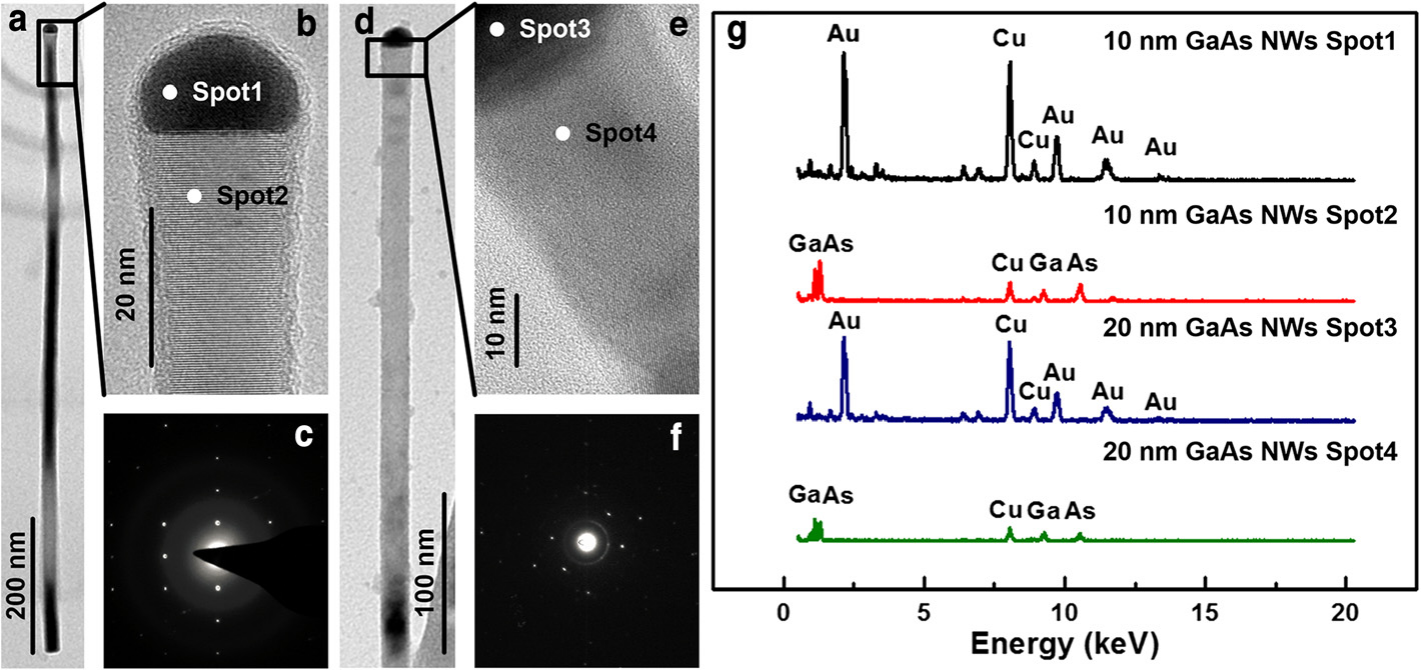
TEM images and EDS analysis of the 10- and 20-nm Au-catalyzed GaAs NWs. **a**–**c** Bright field TEM (BRTEM), high-resolution TEM (HRTEM), and selected area electron diffraction SAED) for 10-nm Au NP-induced NWs whereas (**d**–**f**) are BRTEM, HRTEM, and SAED for 20-nm Au-induced NWs. **g** EDS analysis obtained at the Au NPs and GaAs NW neck region for two sizes of samples and are marked with the corresponding regions in (**b**) and (**e**)

Crystal quality of the GaAs NWs catalyzed by both 10- and 20-nm diameter Au NPs are investigated as shown in Fig. 6. Figure 6a, d are the bright field TEM (BRTEM) images for 10- and 20-nm Au NP-catalyzed NWs displaying good crystallinity of the NWs. There are almost no observable defects across the entire NWs length. Figure 6b, e are the enlarged high-resolution TEM (HRTEM) images displaying the detailed single crystallinity. It can be seen that the 10-nm Au NP-induced NW is along <0001 > direction, whereas the 20-nm Au NP-induced NW is along <111 > direction. Figure 6c, f are the selected area electron diffraction (SAED) patterns for the 10- and 20-nm Au NP-induced GaAs NWs, respectively. It shows that the 10-nm Au NP-catalyzed NWs have a wurtzite crystal structure whereas the 20-nm NWs have a zinc-blende structure. A similar conclusion has been witnessed on other randomly selected NWs on the same sample. It has been reported that wurtzite structure occurs at high liquid supersaturation in the Au catalyst-NWs interface whereas zinc-blende is favored with low liquid supersaturation during VLS growth [16]. According to the Gibbs-Thomson effect, supersaturation is inversely proportional to the diameter of the catalyst. With smaller diameter of Au NPs, liquid supersaturation is higher compared with larger diameter catalyst [24]. Therefore, wurtzite crystal structure was observed for 10-nm Au NP-catalyzed GaAs NWs and zinc-blende for 20-nm samples. In this way, based on our own experimental results, by tuning the diameter of the Au NPs, the crystal structure of the NWs can be changed. Similar results have also been reported by other researchers [16]. From the energy dispersive spectroscopy (EDS) analysis in Fig. 6g, it is well demonstrated that Ga atoms are expelled from the Au NPs after growth, and there are no indium or tin constituents in the GaAs NWs. At the same time, the EDS spectrum also shows that the Ga/As ratio at the NWs is almost 1 which indicates the stoichiometric composition of the NWs.

## Conclusions

In this study, we use the centrifugation method to deposit Au NPs on ITO surface to induce GaAs NWs growth by MOCVD. Various area densities and uniform Au NPs with narrow size distribution were deposited on ITO by tuning the centrifugation time. Compared with the droplet-only method, density-controllable Au NPs were obtained with the highest area densities of 490 and 120 NP/μm^2^ for 10- and 20-nm diameter Au NPs, respectively. The Au NPs were then used as the catalysts to induce the growth of NWs. Free-standing and uniform GaAs NWs were obtained with almost no kinks and worm-shaped defects of the as-grown NWs. Due to the polycrystalline nature of ITO, no single growth direction was favored, but most of the GaAs NWs stand with large tilt angle with ITO surface. The density of the NWs were successfully tuned by the area density of the Au NPs. It was also discovered that the growth rates of the GaAs NWs for both 10 and 20 nm were reduced when the density rose. This was attributed to the competition of the available adatoms on the ITO surface. To clarify, the NWs spacings were calculated using the area density of the Au NPs. Within 4 min of centrifugal deposition of Au NPs, for both 10 and 20 nm, the NWs spacings were larger than the diffusion length of Ga adatoms, and the reduction of growth rates were minor. However, when the centrifugal time was larger than 4 min, the reduction of the NWs growth rate became obvious. The crystal quality of the GaAs NWs was also characterized with almost no observable planar defects. It was demonstrated that 10-nm Au NP-catalyzed NWs possessed a wurtzite structure due to the high supersaturation whereas a zinc-blende structure was observed for 20-nm Au NP-catalyzed NWs. The received GaAs NWs on ITO with free-standing and uniform morphology and high crystal quality with tunable area density demonstrated great potential application in a hybrid solar cell.

## Abbreviations

BRTEM: Bright field TEM
EDS: Energy dispersive spectroscopy
FESEM: Field emission scanning electron microscopy
FETs: Field-effect transistors
HRTEM: High-resolution TEM
ITO: Indium tin oxide
MOCVD: Metal organic chemical vapor deposition
NPs: Nanoparticles
NWs: Nanowires
PDDAC: Polydiallyldimethylammonium chloride
RPM: Revolutions per minute
SAED: Selected area electron diffraction
TBAs: Tertiarybutylarsine
TEM: Transmission electron microscopy
TMGa: Trimethylgallium
VLS: Vapor–Liquid–solid
